# A Preoperative Assessment of Significant Coronary Stenosis Based on a Semiquantitative Analysis of Coronary Artery Calcification on Noncontrast Computed Tomography in Aortic Stenosis Patients Undergoing Aortic Valve Replacement

**DOI:** 10.1097/MD.0000000000002906

**Published:** 2016-03-07

**Authors:** Ji-Won Hwang, Sung Mok Kim, Sung-Ji Park, Eun Jeong Cho, Sans-Chol Lee, Yeon Hyeon Choe, Seung Woo Park

**Affiliations:** From the Department of Medicine, Division of Cardiology (J-WH, S-JP, S-CL, SWP); Department of Radiology (SMK, YHC); Cardiovascular Imaging Center, Heart Vascular Stroke Institute, Samsung Medical Center, Sungkyunkwan University School of Medicine, Seoul (SMK, SJP, S-CL, YHC, SWP); and Division of Cardiology, Department of Medicine, National Cancer Center, Goyang, Korea (EJC).

## Abstract

Invasive coronary angiography (ICA) is the recommended assessment for coronary artery disease in patients undergoing elective aortic valve replacement (AVR). Noncontrast computed tomography (CT) is useful for evaluating lung lesions and calcifications at the cannulation site of the ascending aorta. The purpose of this study was to evaluate the role of noncontrast CT in the visual assessment of coronary artery calcification (CAC) in patients undergoing AVR.

We retrospectively identified patients with significant aortic stenosis (AS) who were referred for AVR between January 2006 and December 2013. Among these, we included 386 patients (53.6% males, 69.2 ± 8.4 years) who underwent both noncontrast CT and ICA. Significant coronary artery stenosis (CAS) in the ICA was defined as luminal stenosis ≥70%. The 4 main coronary arteries were visually assessed on noncontrast CT and were scored based on the Weston score as follows: 0, no visually detected calcium; 1, a single high-density pixel detected; 3, calcium was dense enough to create a blooming artifact; and 2, calcium in between 1 and 3. Four groups were reclassified by the sum of the Weston scores from each vessel, as follows: noncalcification (0); mild calcification (1–4); moderate calcification (5–8); and severe calcification (9–12). Receiver-operating characteristic (ROC) analysis was generated to identify the cutoff Weston score values for predicting significant CAS. Diagnostic estimates were calculated based on these cutoffs.

In the ICA analysis, 62 of the 386 patients (16.1%) had significant CAS. All patients were divided into 4 groups. The noncalcification group had 97 subjects (Weston score 0), the mild degree group had 100 (2.6 ± 1.0), the moderate calcification group had 114 (6.6 ± 1.1), and the severe calcification group had 75 (10.7 ± 1.1). The prevalence of significant CAS in the noncalcification, mild, moderate, and severe groups was 1% (1/97), 5% (5/100), 24% (27/114), and 39% (29/75), respectively. The group with CAS had significantly more CAC than the group without CAS (8.37 ± 2.93 vs 4.01 ± 3.75, *P* < 0.001). The cutoff value (by Weston score) for predicting significant CAS is ≥5 (sensitivity 90.3%, specificity 59.0%, positive predictive value 29.6%, and negative predictive value 97%).

The degree of CAC detected on noncontrast CT can help to predict significant CAS in AS patients who are referred for AVR. For the clinicians, the visual assessment of CAC on noncontrast CT was easy and useful for estimating CAS. Therefore, ICA should be recommended to selective patients based on patients’ CAC and Weston scores during the preoperative evaluation for elective AVR.

## INTRODUCTION

Preoperative assessment of coronary artery disease (CAD) is essential in patients with significant aortic valve stenosis (AS) who are referred for aortic valve replacement (AVR). This assessment helps to determine the need for additional coronary artery bypass grafting (CABG) in patients with severe atherosclerosis, such as men older than 40 years and postmenopausal women.^1,2^ Aortic stenosis and atherosclerosis share several risk factors with CAD.^3–6^ Valvular heart disease was once overwhelmingly caused by rheumatic factors; however, its etiology has shifted toward degenerative factors like CAD.^7,8^

Invasive coronary angiography (ICA) is the criterion standard for diagnosing significant coronary artery stenosis (CAS). The current guidelines include a class IC recommendation to perform ICA before AVR in patients with symptoms of angina, objective evidence of ischemia, decreased LV systolic function, history of CAD, or coronary risk factors including men older than 40 years and postmenopausal women.^1,2^ However, ICA carries a small but non-negligible risk of both major and minor complications including subclinical systemic emboli, stroke, arterial puncture, angina, coronary artery dissection, myocardial infarction, and reactions to contrast media (0.3%).^9^ The operator who performs the ICA is also exposed to radiation.^10^

Noncontrast CT is a necessary and practical method for evaluating calcification at the cannulation site of the ascending aorta before AVR. Generally, the degree of coronary artery calcification (CAC) can be accurately estimated based on electrocardiogram (ECG)-gated calcium-scoring computed tomography (CSCT) images. Previous studies suggested that the CAC score (in the detection of CAD) obtained from ungated low-dose CT (LDCT) was comparable with that measured with ECG-gated CSCT. These studies revealed a good correlation between the CAC scores obtained using 2 CT scan protocols.^11,12^ CAC is a well-known marker for significant CAS, which is also evaluated by noncontrast CT.^13–15^

In clinical practice, both noncontrast CT and ICA are performed in AS patients before AVR. If the CAC detected on noncontrast CT predicts significant CAS, ICA should only be performed in select patients before AVR. We conducted a retrospective study to evaluate the role of visual CAC assessments of noncontrast CT for predicting significant CAS in AS patients undergoing AVR.

## METHODS

### Study Population

Patients were screened for inclusion in this study if they had been diagnosed with moderate-to-severe AS and were scheduled to undergo elective AVR (with or without CABG) between January 2006 and December 2013. Severe AS was defined as aortic valve area <1 cm^2^ based on the recommendations of the American Society of Echocardiography.^16^ A total of 386 severe AS patients who underwent both ICA and noncontrast CT were enrolled.

We retrospectively reviewed the medical records of the enrolled patients. Additional information was collected including baseline demographic characteristics, underlying medical history, laboratory findings, EuroSCORE,^17,18^ echocardiographic data, ICA findings, and surgical treatment. We also determined whether the enrolled patients had undergone AVR with, or without, simultaneous CABG. This study was approved by the institutional review board at our institution. Informed consent was waived.

### The CT Protocol and Image Analysis

All patients underwent cardiac CT using a dual-source CT system (SOMATOM Definition Flash, Siemens Medical Solution, Forchheim, Germany) with a 2 × 64 × 0.6 mm detector collimation. The CT was collected using the *z*-axis flying focal spot technique, resulting in 2 × 128 sections. A noncontrast CT scan was acquired according to the following parameters: 280 msec gantry rotation time, 100-kV tube potential, and real-time tube current modulation with 250 reference mAs, according to the precise shape of the patient's body (Automatic tube current modulation in the x, y, z direction; CARE DOSE 4D, Siemens Medical Systems). CT images were reconstructed using 2.0-mm section thickness and a 2.0-mm reconstruction increment using a soft kernel (B31f). Because we used previously obtained data, we compare the ICA with noncontrast and non-ECG-gated CT and used the Weston score based on the reference,^13^ not the Agatston score.

**FIGURE 1:**
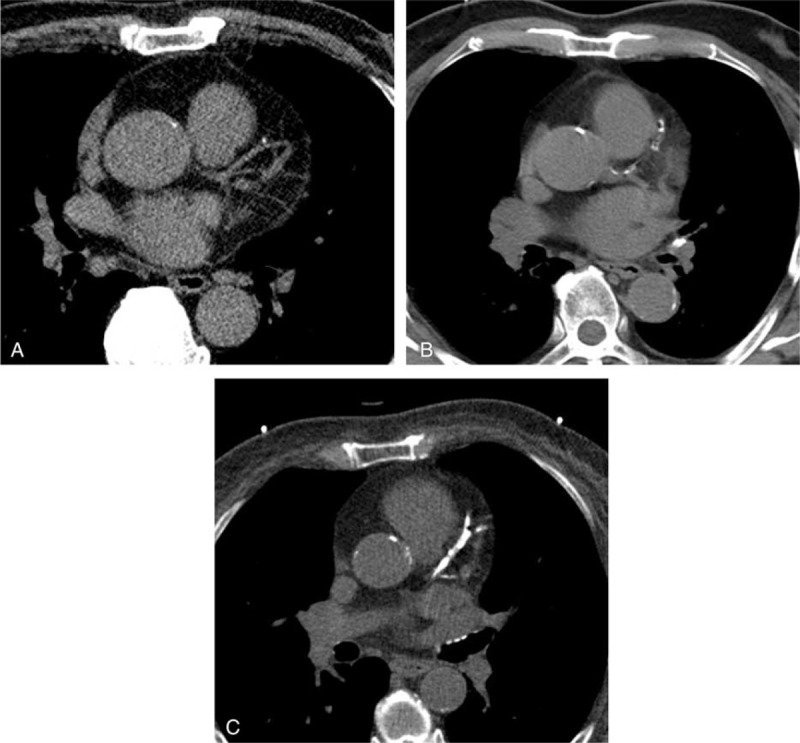
Coronary artery calcification with a Weston score 1–3 on an axial standard image on noncontrast CT. (A) A punctate fossae of increased attenuation at the left anterior descending (LAD) coronary artery graded as a Weston score of 1. (B) A scattered (nonblooming) calcified plaque involving the LAD coronary artery graded as a Weston score of 2. (C) A dense calcified plaque with blooming at the LAD coronary artery graded as a Weston score of 3.

The noncontrast CT examinations were analyzed visually using mediastinum soft tissue window settings. The Weston score was estimated for each major coronary vessel (the left main trunk, the left anterior descending artery, the left circumflex artery, and the right coronary artery). It was scored as follows: 0, no visually detected calcium; 1, a single high-density pixel is detected; 3,calcium is dense enough to create a blooming artifact; and 2, calcium between 1 and 3 (Figure 1). The Weston score was calculated by the sum of the calcium scores for each vessel (range 0–12).^13^ A single cardiac imaging radiologist (SMK) with 11 years of experience in cardiothoracic CT interpretation evaluated all of the cases.

### ICA

ICA was performed using standard techniques based on the operator's discretion. All baseline coronary angiograms were reviewed and analyzed at the angiographic core laboratory (Heart Center, Samsung Medical Center, Seoul, Korea) with an automated edge-detection system (Centricity CA 1000, GE, Waukesha, WI) using standard definitions.^19^ CAS was considered to be significant if ≥70% of the luminal diameter was constricted (compared with the reference) by visual estimation on at least 2 orthogonal views.^20^ Any suspected, significant CAS was confirmed by ICA.

### Statistical Analysis

Quantitative variables are reported as the means and standard deviations. In the univariate analysis, student *t* tests were used to compare continuous variables and *χ*^2^ tests were used for categorical variables. Multiple logistic regression analysis was used to identify independent factors that are associated with significant CAS. The Bonferroni correction was applied to compare the degree of CAC with the ICA findings. To ensure an overall type I error rate of 5%, an adjusted *P* value of 0.05/6�=�0.008 was considered significant. In addition, receiver-operating characteristic analysis was generated to identify cutoff values of the Weston score (defined as those with the greatest sum of sensitivity and specificity).

All analyses were conducted using SPSS (version 20.0, SPSS Inc, Chicago, IL), and Medcalc (version 9.6). *P* values <0.05 were considered statistically significant.

## RESULTS

### Baseline Characteristics

A total of 386 patients who underwent both ICA and noncontrast CT were referred for elective AVR, and their baseline clinical characteristics and TTE findings are shown in Table 1.

**TABLE 1:**
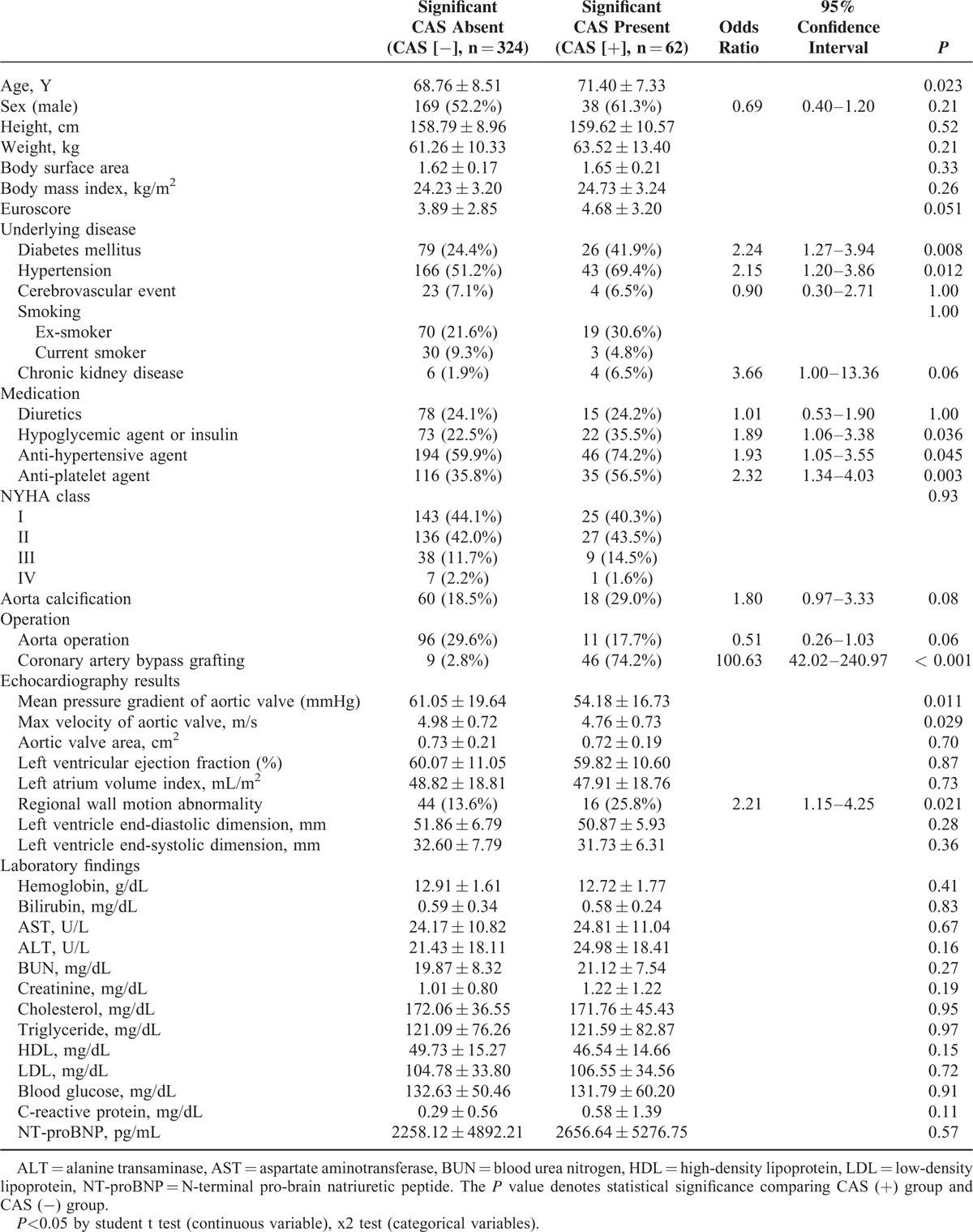
Baseline Clinical Characteristics

Sixty-two patients were diagnosed with significant CAS (stenosis up to 70% in any major vessel). The significant CAS group (CAS [+] group), therefore, had 62 patients (71.40 ± 7.33 years). The nonsignificant CAS group (CAS [−] group) had 324 patients (68.76 ± 8.51 years). There was a higher EuroSCORE in the CAS (+) group (4.68 ± 3.20 vs 3.89 ± 2.85, *P* = 0.051) than in the CAS (−) group. The CAS (+) group had a higher prevalence of underlying diabetes mellitus (DM) (*P* = 0.008) and hypertension (*P* = 0.012) than the CAS (−) group. The CAS (+) group used more anti-platelet agents than the CAS (−) group (*P* = 0.003).

### The Weston Score as a Visual Assessment of Coronary Artery Calcification

All patients were divided into 4 groups. There were 97 patients in the noncalcification group (Weston score 0), 100 in the mild calcification group (2.6 ± 1.0), 114 in the moderate calcification group (6.6 ± 1.1), and 75 in the severe calcification group (10.7 ± 1.1). ICA analysis revealed 62 patients (16.1%) with significant CAS. The prevalence of significant CAS in each group was as follows: 1% (1/97), 5% (5/100), 24% (27/114), and 39% (29/75). The degree of CAC was significantly higher in the CAS (+) group than in the CAS (−) group (8.37 ± 2.93 vs 4.01 ± 3.75, *P* < 0.001) (Table 2). In addition, the frequency of significant CAS was significantly different between the 4 groups. The patients with higher grades of calcification (moderate and severe) were most prevalent in the CAS (+) group. The Bonferroni correction revealed statistical significance between the mild and moderate degrees of calcification (*P* < 0.001). The cutoff Weston score for predicting significant CAS is ≥5 (sensitivity 90.3%, specificity 59.0%, positive predictive value 29.6%, and negative predictive value 97%). After adjusting for multiple variables, multiple logistic regression analysis revealed that the CAC grade was the only independent factor for assessing significant CAS (Table 3).

**TABLE 2:**
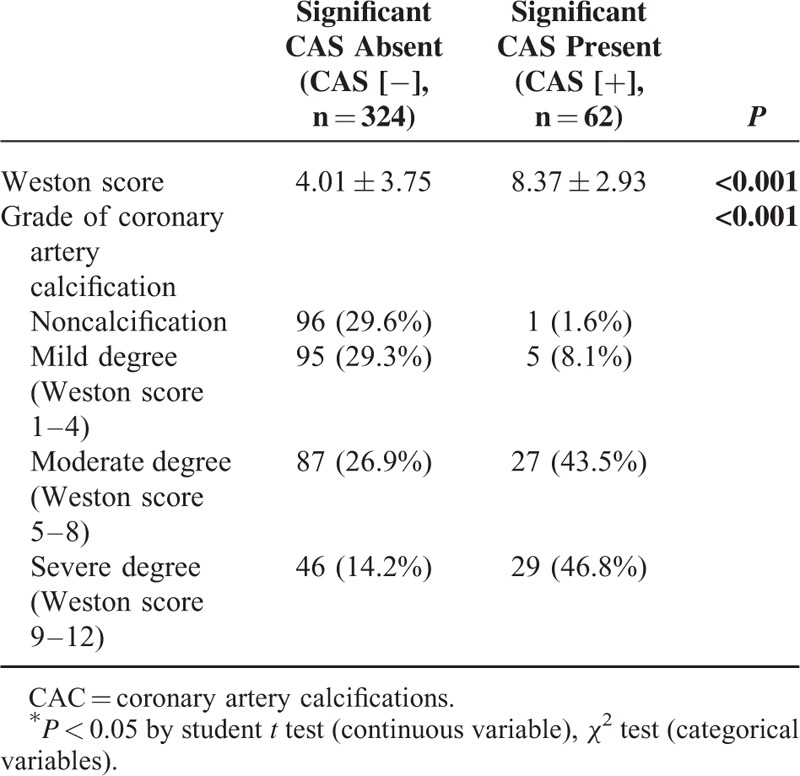
Visual Assessment of the Aortic Valve and CACs in the Aorta on Noncontrast Computed Tomography

**TABLE 3:**
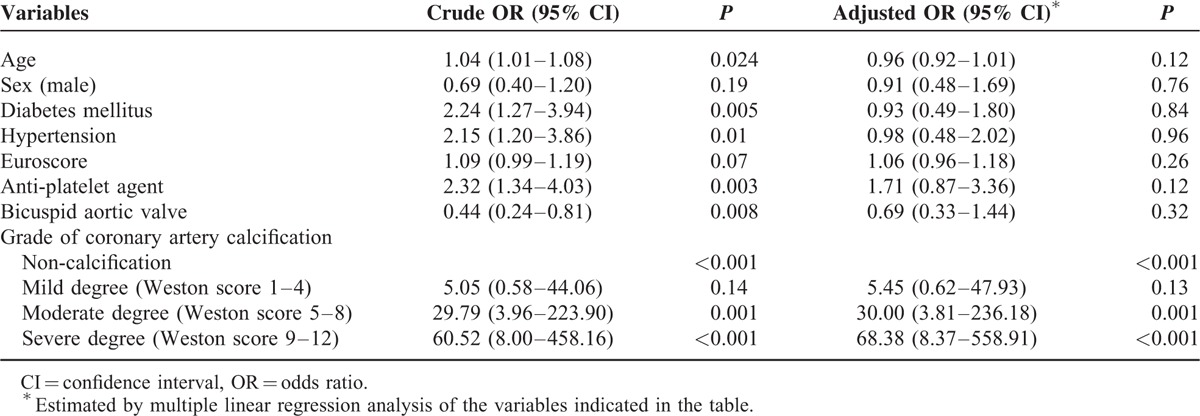
Univariate and Multiple Analyses of the Clinical Factors Associated With Significant Coronary Artery Stenosis in All Patients

### CABG

The 1-vessel disease was defined as luminal stenosis ≥70%. Of 55 patients, 27 patients were diagnosed with 1-vessel disease, 14 patients were diagnosed with 2-vessel disease, 13 patients were diagnosed with 3-vessel disease, and just 1 patient had left main and 3-vessel disease.

**FIGURE 2:**
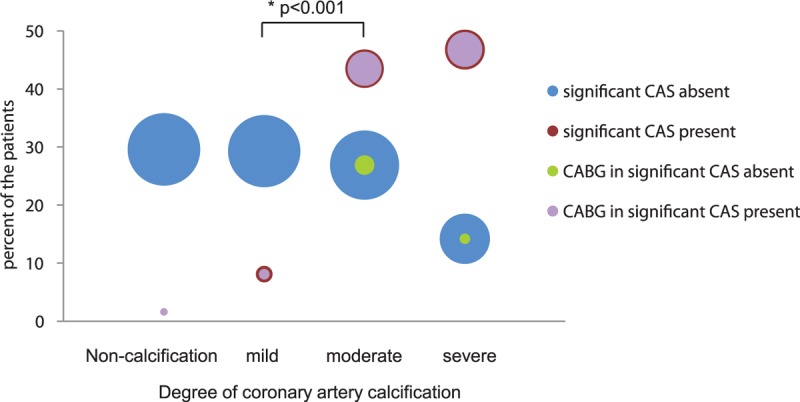
Reclassification of the 4 grades of coronary artery calcification based on the visual assessment of the Weston score in 2 groups with and without coronary artery stenosis (CAS). The number of patients undergoing coronary artery bypass grafting (CABG) in each group and grade. ^∗^The *P* value was estimated using Bonferroni correction (*P* < 0.001).

The need of CABG was decided by the charge physician based on the results of the ICA and the patient's current condition. Among the CAS (−) group (n = 324), CABG was performed in only 9 patients (2.8%) (Table 1). Most patients in the CAS (+) group underwent CABG (Figure 2). Only 3 of 197 patients (2 from the noncalcification group and 1 from mild calcification group) underwent CABG.

## DISCUSSION

The major findings of the present study are the following: visual assessment by CAC on noncontrast CT was sufficient to detect significant CAS in 27 patients with moderate calcification (43.5% among the 62 patients with significant CAS) and 29 patients with severe calcification (46.8%); and higher degrees of CAC on noncontrast CT allowed for better assessments of significant CAS in the pre-evaluation for elective AVR.

### Severe AS and CAD

Patients with severe AS have a relatively high incidence of CAD, ranging between 20% and 50%.^1,5,7,21^ The extent of CAS involvement in patients with significant AS is predictive of the morbidity and mortality associated with AVR, as well as long-term prognosis.^3,5,22–24^ It is critical to evaluate patients for significant CAD before AVR. Noninvasive imaging studies such as low-dose exercise stress echocardiography or single-photon emission computerized tomography (SPECT) or cardiac magnetic resonance imaging were studied for estimating the CAD in severe AS.^25–27^ The current guidelines suggest that ICA is indicated before valve intervention in patients with symptoms of angina, objective evidence of ischemia, decreased LV systolic function, CAD history, or coronary risk factors (including men older than 40 years and postmenopausal women) (Class I, Level of evidence C). Coronary CT angiography can be used to exclude significant CAD in select patients with a low/intermediate pretest probability of CAD (Class IIa, Level of evidence B).^28–31^ ICA is the criterion standard for evaluating significant CAS. However, ICA in patients referred for elective AVR is not only difficult (owing to aortic root dilatation) but also can be of relatively high risk. ICA may result in fragmentation of a calcified aortic plaque and subsequent subclinical cerebral embolism or disabling stroke.^32–34^ However, ICA should still be carried out in all patients before AVR, regardless of the pretest probability of CAD. This is because the presence of angina pectoris has a poor predictive value, and noninvasive tests in general lack accuracy. Our group previously reported that the overall incidence of angiographically significant CAS in severe AS undergoing AVR was 10.6%.^35^ In this study, the incidence of significant CAS was as low as 16.1%. Therefore, ICA before AVR should be considered in patients with multiple risk factors for cardiovascular disease.^35^

With regard to evaluating contrast CT, the Agatston score has been found to be in accord with the results obtained using nonenhanced CSCT.^13,36,37^ The CAC scoring on contrast coronary CT angiography is closely associated with the severity of coronary atherosclerosis and clinical outcomes.^37^ Previous studies have shown that multislice CT with a high negative predictive value as a first method means of ruling out CAD in preoperative assessment is recommended, such as the accurate Agatston score.^20,38,39^ Multislice CT is more useful in the diagnostic work-up of patients at low-to-intermediate risk for CAD.^40–43^ Furthermore, the visual CAC assessment on non-contrast CT provides clinical information, such as the risk of cardiovascular death.^14,44,45^ Noncontrast, cardiac CT is a promising technique for imaging beyond calcification of the coronary tree.^45^ It provides information about intrathoracic anatomy (such as the aortic arch dimension, aortic arch calcification, arterial course, and lung disease). These data points are useful for the cardiac surgeon with regard to cannulation, and cross-clamping sites. In addition, using a more extensive scan protocol with retrospective electrocardiogram-gating for functional and aortic valve analysis, the aortic valve, myocardium, and coronary arteries were simultaneously evaluated.^20,46,47^ Therefore, in this retrospective study, we tried to estimate that noncontrast CT might be a useful method for the visual assessment of CAS, although a noncontrast CT scan was performed to evaluate calcifications at cannulation sites and concomitant lung disease. In other studies of non-contrast CT, ECG-gated coronary CT angiography was used as the reference standard for CAD.^12–15^ However, we used the ICA as the reference standard of CAD by ICA.

Not all patients with >50% stenosis in coronary artery should undergo bypass surgery. It was difficult to decide to perform bypass surgery in patients with CAS of >50% and <70%. In addition, it was also difficult to discriminate symptoms of AS from CAD for patients with significant AS. We therefore referred to the work of Larsen et al,^20^ who defined significant CAS as over 70% in a study population similar to ours.

As the major etiology of valvular heart disease has shifted,^7^ the treatment strategy for severe AS has also changed. Older patients with severe AS and CAD can be included in the high perioperative risk group. The standard treatment for patients with severe AS and CAD is surgical AVR with simultaneous CABG. However, interventions that combine AVR and CABG are associated with higher postoperative mortality than AVR alone.^48^ In the largest study to date, Goel et al^49^ found that PCI did not increase the risk of short-term mortality or procedural complications in patients with severe AS compared with those without AS. Therefore, ICA should be reconsidered with regard to the preoperative evaluation when assessing CAD.^50,51^

Using noncontrast CT as the first preoperative assessment actually reduced the effective radiation dose and cost compared with patients who underwent ICA alone before AVR. In addition, the initial evaluation of noncontrast CT can reduce the contrast compared with the combination of coronary CT angiography and ICA. For instance, despite using coronary CT angiography, ICA should also be performed in some patients with significant calcium because of the blooming artifact. This allows for the accurate assessment of CAS. Because these cases require a double dose of contrast, coronary CT angiography was not performed routinely. This result may be particularly applicable in patients with existing azotemia or contrast allergy. Our results suggest that after evaluating noncontrast CT, ICA should be performed in patients with high Weston scores.

Visual assessment of CAC by the Weston score may be a helpful, initial preoperative evaluation for predicting significant CAS. In this study, 43 of the 56 patients with moderate and severe CAC underwent CABG. The Weston score may be useful for the visual assessment of CAC, and may be appropriate in noncontrast CT. Visual assessment of CAC by the Weston score is an easy and simple method that clinicians can use.

## LIMITATIONS

This study has several limitations. For one, it was a nonrandomized, retrospective, and observational in design. These data, which compared ICA with a visual assessment of CAC on noncontrast CT, were based on a single center, so there was a selection bias. In addition, the sample size was relatively small. These factors may have significantly affected the results secondary to confounding. We could not evaluate the Agatston score in ECG-gated CT because we just retrospectively estimated calcification from the previously collected data.

Therefore, the study did not have sufficient power to reveal the significance or superiority of noncontrast CT. This study may have also been affected by inclusion bias, as it only enrolled patients who were scheduled for elective valve surgery. In the future, large-scale, prospective randomized controlled trials are needed to clarify the role of non-contrast CT compared with ICA in patients undergoing elective AVR.

The coronary arteries may still be obstructed, even if there is no CAC. Similarly, a positive CAC score is not a direct indicator of significant CAS. Preoperative assessment of the coronary arteries cannot be limited to calcification evaluation. Moreover, noncalcified plaques (more specifically low-attenuation plaques) were more prone to produce hemodynamically significant coronary stenosis (as defined with the use of fractional flow reserve).^52^ The isolated evaluation of coronary calcification is and will remain too limited to be accepted for the purpose of pre-operative evaluation. Furthermore, the degree of CAC is not sufficient to exclude CAD in symptomatic patients and is often followed by CT angiography or ICA. Additional studies are needed to determine the optimal indication for performing ICA, as associated with the degree of CAC.

## CONCLUSION

In patients with AS who are referred for AVR, the degree of CAC detected on noncontrast CT may be useful for evaluating CAS. The visual assessment of CAC on noncontrast CT was an easy and useful method for clinicians evaluating CAS.
